# Functional Redundancy Instead of Species Redundancy Determines Community Stability in a Typical Steppe of Inner Mongolia

**DOI:** 10.1371/journal.pone.0145605

**Published:** 2015-12-23

**Authors:** Saruul Kang, Wenjing Ma, Frank Yonghong Li, Qing Zhang, Jianming Niu, Yong Ding, Fang Han, Xiaoli Sun

**Affiliations:** 1 Ecology, School of Life Sciences, Inner Mongolia University, Hohhot, China; 2 Sino-US Center for Conservation, Energy, and Sustainability Science, Inner Mongolia University, Hohhot, China; 3 Grassland Research Institute of Chinese Academy of Agricultural Sciences, Hohhot, China; 4 Inner Mongolian Meteorological Bureau, Hohhot, China; University of Oklahoma, UNITED STATES

## Abstract

**Background:**

The redundancy hypothesis predicts that the species redundancy in a plant community enhances community stability. However, numerous studies in recent years questioned the positive correlation between redundancy and stability.

**Methodology:**

We explored the relationship between the species redundancy, functional redundancy and community stability in typical steppe grassland in Northern China by sampling grassland vegetation along a gradient of resource availability caused by micro-topography. We aimed to test whether community redundancy enhanced community stability, and to quantify the relative importance of species redundancy and functional redundancy in maintaining community stability.

**Results:**

Our results showed that the spatial stability of plant community production increases with increased supply of soil resources, and the functional redundancy instead of species diversity or species redundancy is correlated with the community stability. Our results supported the redundancy hypothesis and have implications for sustainable grassland management.

## Introduction

The relationship between species diversity and ecosystem stability and its maintenance mechanism has been one of the hotspots in ecology research [1–3]. Human-driven loss of diversity might reduce ecosystem stability [4], but the generalizations remain unclear. Both positive [1–3, 5] and negative correlations [6] as well as nonlinear relationships [7] are found between diversity and stability. For instance, diversity can positively affect stability of community biomass [8], but, it can decrease the population stability [5]. As a consequence, ecosystem stability could be negatively affected if diversity destabilizes the population stability [9].

Many hypothesized mechanisms for the maintenance of species diversity and ecosystem function have been proposed; including, but not limited to, the diversity-stability hypothesis [2], rivet-popping hypothesis [10], species redundancy hypothesis [11], idiosyncratic hypothesis [12], and keystone species hypothesis [13]. Among these, the redundancy hypothesis proposed by Walker [11] has increasingly become an important concept in understanding the ecological values of biodiversity. This hypothesis proposes that in a functional group which contains many species, the species often manifest functional redundancy. That is, some species present similar or identical features and these species show asynchronous responses to environmental changes, or display a phenomenon of time niche differentiation [11, 14], which helps maintain system stability during disturbance [15–17]. However, the accuracy of the redundancy hypothesis was doubted by some scholars [6], thus was not widely accepted [18]. Four points of contention arise about the redundancy hypothesis. The first point arises from the different concepts of redundancy. Mori et al. [6] proposed that low temporal redundancy and trait-based redundancy were two distinct aspects of redundancy, and they maintain that redundancy should be evaluated using multiple factors in addition to functional effect traits to ensure more accurate characterization of ecosystems. The second point is the different calculations of redundancy, either at species level, obtained by performing statistics of species richness in the group [18, 19], or at functional group level. Bello et al. [20] proposed a new method to calculate the functional redundancy, and believed that the functional redundancy is a part of species diversity that is not manifested with functional diversity, that is, the functional redundancy is the difference between species diversity and functional diversity. Third, the different viewpoints might partly originate from variation in the calculation of stability which is, central to any stability-related hypothesis, such as the community structure stability (e.g., community similarity in Pillar et al. [21] and in Gordan [22]), and functional stability (e.g., temporal stability (S) in Tilman et al. [5]). The variability of productivity is widely used to measure stability however different calculation methods may lead to different results [5]. Fourth, the stability of the same ecosystem may show different features at different scales [23]. For example, a forest fire may reduce the stability of a coniferous forest in western United States, although fire is an important ecological factor for maintaining the ecological stability at a regional level [24, 25]. Meanwhile, the linkage between the ecological factors and stability also changes with scale and impact factors [26]. All these points need to be considered to determine the most applicable redundancy hypothesis for the study of relationship between diversity and stability.

While the large scale relationships between diversity and ecosystem functions in Inner Mongolia grassland has been investigated [27–29], studies at a small scale are lacking. Topography is the most important environmental factor within a small-scale landscape where the re-distribution of resources in space, affects the diversity and ecosystem functions. Here we analyze the changes of the diversity and community stability of the typical steppe grassland in Inner Mongolia, as well as their relationship under different resource supply rates caused by micro-topography. We aim to answer the following questions: (1) what type of relationship exists between the diversity and ecosystem stability in a typical Inner Mongolia steppe; (2) does the redundancy hypothesis hold (redundancy enhancing stability) in this region and (3) which of species redundancy or functional redundancy is more important in maintaining the stability. These studies may provide scientific basis for restoration of degraded grassland in addition to understanding the relationships between diversity and stability.

## Methods

### Study Areas

The sample area is located in the Ecology Research Base of Inner Mongolia University (within 44.26°N—44.28°N and 116.32°E—116.35°E). The region has a continental climate, with a mean annual temperature of 0.88°C(1983–2005). Mean annual precipitation was 338 mm (coefficient of variance (CV) = 22%), of which 85% occurred from May through to September. The growing season is about 150 days long, with 200–235 frost free days. The land is hilly and the soil is calcic kastanozems and the vegetation is typical steppe. A vegetation gradient occurs along a resource gradient associated with different slope positions; *Leymus chinensis (Trin*.*) Tzvel*. dominates the community in fertile lowland, while two *Stipa* species (*S*. *grandis P*. *Smirn* and *S*. *krylovii Roshev*.) dominate the communities on the slope and top of the hills where there is a poor resources.

### Vegetation survey

Vegetation was surveyed in plant biomass peak period in this region (early August 2013) using a plot-transect method. Seven line transects perpendicular to the slope were evenly set from the top of hills to the lowland, i.e., the seven line transects were aligned in an ascending order from top to bottom. The length of each line transect was 31 m, and 1 m x 1 m quadrates were set on each line transect with an interval of 1 m, each line transect containing 16 quadrats, totally 112 quadrats. The height of plant vegetative and reproductive layers and the number of plants (abundance) in each quadrat were recorded by species, and aboveground biomass was determined by clipping the plants and obtaining fresh and dry weights (dried to constant weight at 65°C, about 48 hour). The frequency of each species in each transect was calculated as its presence in the 16 quadrats of one transect. The functional traits of each species that appeared in the 16 quadrats were determined by sampling 6 well-grown plants in the corresponding transect. The measured functional traits include plant height (H), number of leaves, leaf area (LA, using Portable Laser Leaf Area Meter), and leaf dry weight (LDW, oven-dried at 65°C). The average aboveground weight (W), specific leaf weight (SLW) and specific leaf area (SLA) of individual plants were calculated. The five plant functional traits: W, H, LDW, LA and SLA were examined in this analysis.

### Soil nutrition test

Soil samples were collected from three drills to 30 cm in depth within each quadrat. The mixed sample of the 3 drills were brought to laboratory to determine the content of total N, total P, available N (NH_4_^+^ and NO_3_^-^), available P (A-P), and organic C. Total N was determined using selenium-cupric sulfate (CuSO_4_)-potassium sulfate (K_2_SO_4_)-heating digestion method; total P was measured by alkali fusion-Mo-Sb colorimetric method; available N was measured by Kjeldahl nitrogen determination method, and available P was measured by sodium bicarbonate (NaHCO_3_) leaching-Mo-Sb colorimetric method; and organic C was measured by potassium dichromate (K_2_Cr_2_O_7_) heating oxidation method [30].

### Data analysis

Calculation of species diversity, community stability and redundancy:

Species diversity (SD) was calculated using Shannon-weaver species diversity index since it represents the complexity of the community [31].

$$SD=\sum_{i=1}^{s}|p_{i}\text{lg}\left(p_{i}\right)|$$ (1)

where *p*_*i*_ refers to the relative abundance of species *i* in the community.

Community stability was calculated using reciprocal of variation coefficient of aboveground biomass in 16 quadrats at each line transect (*S* = *υ* ÷ *σ*) [5].

Community redundancy was calculated based on two main methods: The first is based on the number of species that were functionally redundant in community. As the keystone or dominant species occupy a large proportion of community function, and the redundant species possess few functional properties [11, 32], the species redundancy was calculated based on the frequency of species which were redundant to the productivity of the functional group. The species in the communities were divided into 5 functional groups according to their life-forms: shrub and half shrub (SS), perennial bunchgrass (PB), perennial rhizomatous grasses (PR), perennial forbs (PF) and annual and biennial herbs (AB) [33]. The species in each functional group were arranged in descending order of their productivity contribution rate, then the species that made up the last 20% of contribution rate in a group were considered as redundant species for that group, and the average number of redundant species of the five functional groups in each transect was considered as the species redundancy of the community.

The second method is according to Bello et al [20] who believed that the functional redundancy is the part of the species diversity in the community that has not been explained by functional diversity. Therefore, functional redundancy (FR) is the difference between the functional diversity (FD) and the species diversity (SD) [20, 21]. That is,

FR=SD−FD (2)

where *SD* is calculated by eq 1, and *FD* is represented by Rao coefficient [34].

$$FD=\sum_{i=1}^{s}\sum_{j=1}^{s}d_{ij}p_{i}p_{j}$$ (3)

where *d*_*ij*_ is the functional feature distance between species *i* and species *j*, *p*_*i*_ and *p*_*j*_ are ratios of individual numbers of species *i* and species *j* over the individual number of overall species in the community, respectively. *S* is the overall species number in the community.

### Statistical analysis

Statistical analyses were performed using SPSS 17.0 software. First, the variation tendencies of the species diversity and community stability along the gradient of resources supply (at different slope positions) were analyzed and their causal relationship was identified using regression analysis. Then, the relationships between the species diversity and community stability, as well as among species redundancy, functional redundancy and community stability were examined using regression analysis to elucidate the role of species diversity, species redundancy and functional redundancy in community stability.

## Results

### Soil resources supply increased with the descending order of the slope positions

Soil fertility indicators total N, total P, available N, and organic C in seven transacts showed an increasing trend with decreasing slope position from the top to the bottom of hills, except the available P which showed no spatial change down the slope (Table 1). That is, soil fertility or resource supply increased along the gradient from the top to the bottom of the hills.

**Table 1:** Soil fertility indicators (AN—available N; AP—available P; SOC—soil organic carbon), species diversity (SD), species redundancy (SR), functional redundancy (FR), and community stability (S) in seven transects.

	No. of samples	Total N (mg/100 g)	Total P (mg/100 g)	AN. (mg/100 g)	AP. (mg/kg)	SOC (g/100 g)	SD	FR	SR	S
**Tran 1**	16	114.59	27.71	9.63	3.83	0.97	0.48	2.23	0.25	2.52
**Tran 2**	16	111.92	28.35	10.00	3.68	0.89	0.44	1.30	0.25	3.13
**Tran 3**	16	114.16	28.44	10.1	2.6	0.91	0.48	1.59	0.29	3.61
**Tran 4**	16	113.43	29.01	9.98	2.02	0.94	0.60	1.91	0.42	5.22
**Tran 5**	16	119.26	28.95	10.28	1.73	0.98	0.49	1.94	0.44	5.93
**Tran 6**	16	152.23	35.57	12.44	2.24	1.21	0.68	1.94	0.38	6.09
**Tran 7**	16	248.64	54.59	15.82	2.74	1.57	0.73	2.35	0.43	5.94

The transect 1 to 7 were ordered according to their position on the slope from the top to the bottom of the hill.

### The change of diversity and stability with the soil resource supply

**Fig 1:**
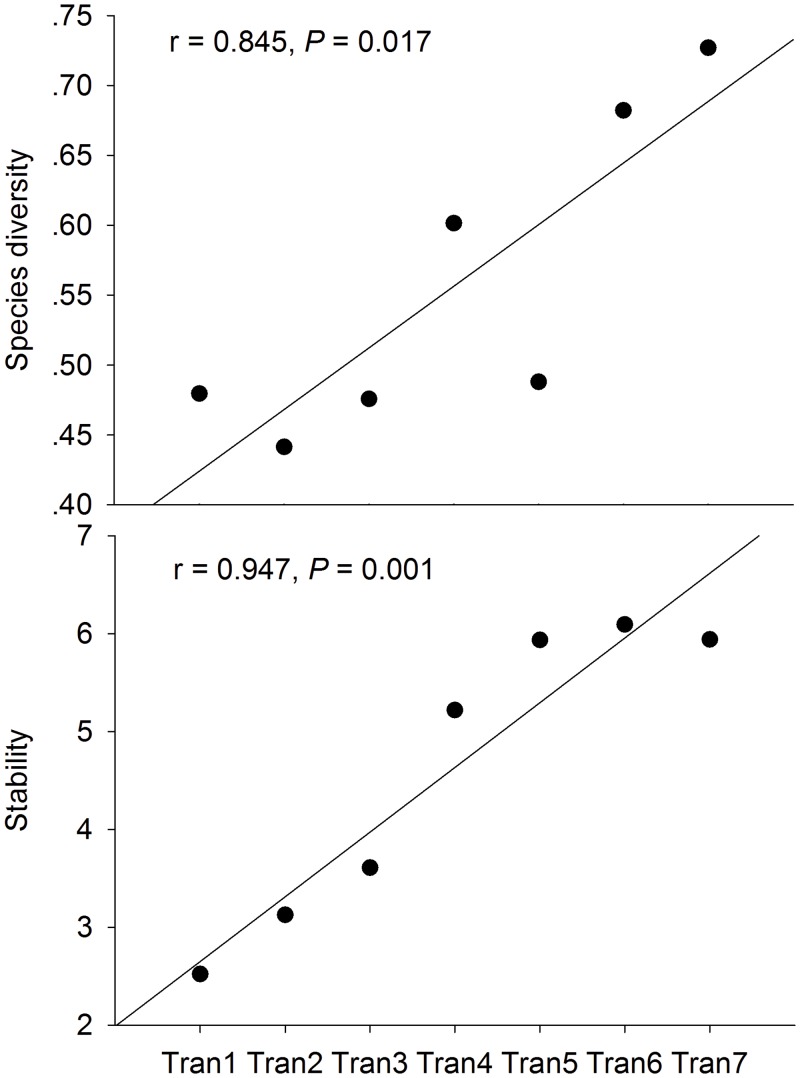
Both species diversity and community stability increase with increasing resources availability.

**Fig 2:**
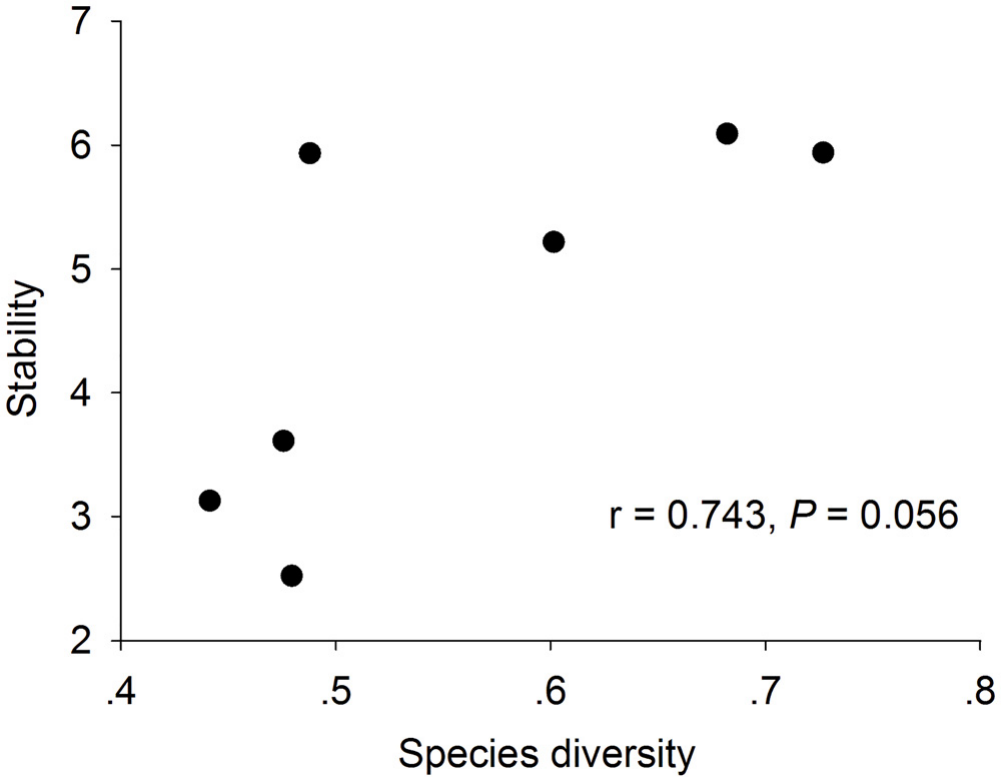
Trend in community stability with species diversity.

We used regression analysis to verify the change of species diversity and stability of aboveground biomass with slope position, and the change of stability with species diversity. The results showed that species diversity and stability both increased with decreasing slope positions (Fig 1: r = 0.845, *p* = 0.017, r = 0.947, *p* = 0.001 respectively; Table 1), i.e., with the increase of resources supply. Further species diversity had no causal effect on stability (r = 0.743, *P* = 0.056) (Fig 2).

### Relationship of two redundancies

**Fig 3:**
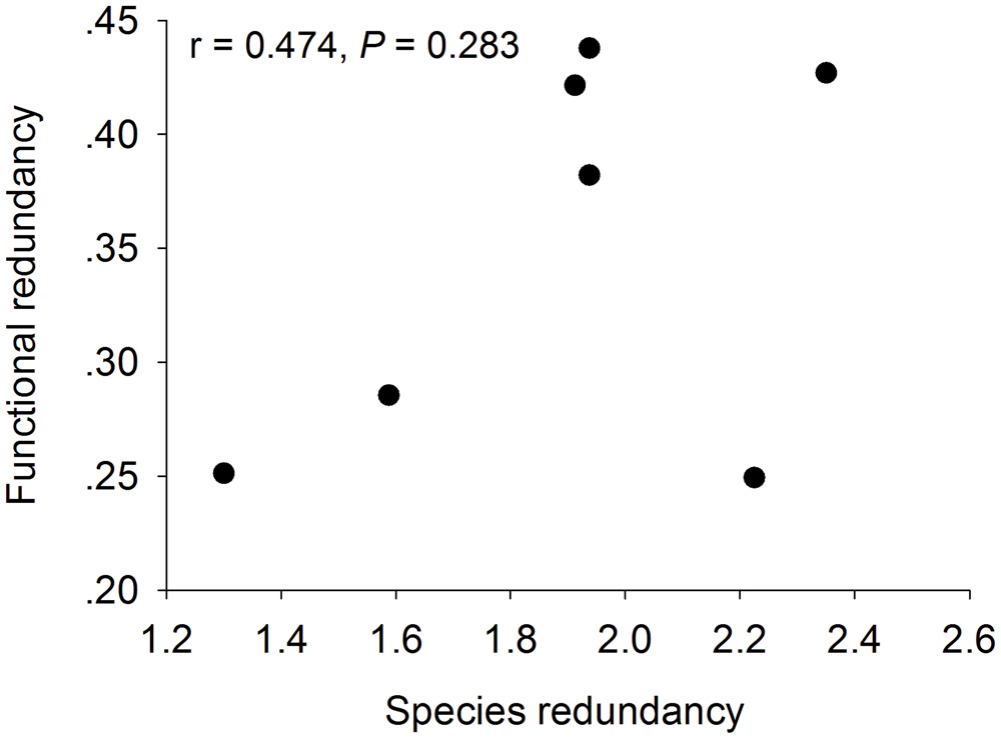
Species redundancy and functional redundancy showed no relationship.

We used regression analysis to demonstrate the relationship between two redundancies. The regression analysis of species redundancy and functional redundancy revealed that species redundancy had no causal effect on functional redundancy (r = 0.474, *P* = 0.283) (Fig 3).

### Relationship between redundancy and stability

**Fig 4:**
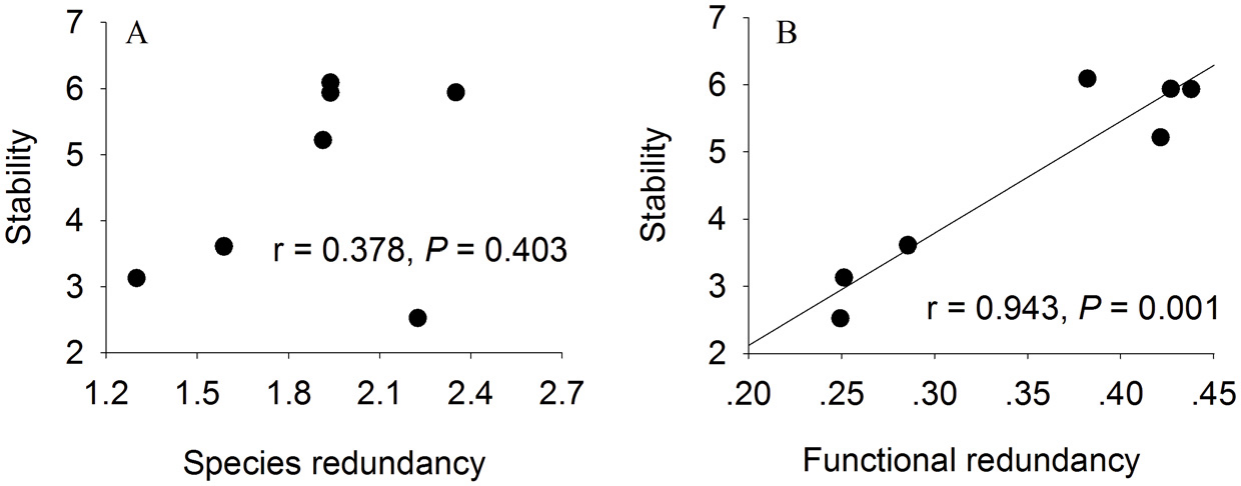
Trend in stability with species redundancy (panel A) and functional redundancy (panel B).

We also used regression analysis to illustrate whether the species redundancy and functional redundancy stabilize the communities’ productivity or not. The results of regression analysis for redundancies and stability showed that functional redundancy was correlated with community stability (r = 0.943, *P* = 0.001), but not species redundancy (r = 0.378, *P* = 0.403) (Fig 4A and 4B). Community stability increased with an increase in functional redundancy.

## Discussion

### Species redundancy and functional redundancy are two different features of community redundancy

The diversity-stability relationship is a subject of much debate [1, 35]. The redundancy hypothesis, as presented by Walker [11], is an important hypothesis in theoretical exploration of the relationship between diversity and stability [15]. The redundancy hypothesis proposes that some species may be ready at all times to expand their ecosystems ‘jobs’ to compensate when neighboring species go extinct [10]. At the heart of the concept is the idea that species may be segregated into functional groups, and that species in the same functional group are considered to be more redundant than species in different functional groups [36], this means that, ecosystems can remain stable in the face of disturbance [37]. The question is, how much redundancy should ecosystems have to guarantee the ecosystem stability? Scholars have applied a variety of experimental methods to measure community redundancy. An earlier method for calculating community redundancy used species richness of the functional group to represent the redundancy [11, 15, 18, 38]. Community redundancy was also indirectly expressed using curves representing linkage between species diversity and functional diversity. If a curve of a group of communities presents a saturation state, it is believed that the community at that point on the curve contains redundancy [19]. If the curve presents a positive linear correlation, it is believed that this group of communities does not contain redundant communities [39]. This method has been often used to test the loss of community functional diversity with different utilization patterns and intensities of land use [40]. Subsequently, Bello et al. [20] proposed a new method to calculate community redundancy, wherein they believed that the community redundancy is the part of species diversity that has not been expressed by functional diversity. Therefore, the community redundancy can be expressed as the difference between the species diversity and the functional diversity.

We have calculated the community redundancy using the first and the third methods mentioned above for the redundancy of communities located in different slope positions in the sample area separately, and found no correlations between the redundancies obtained with these two methods (P > 0.05). The reason for this might be that a small number of keystone species or dominant species account for a large function proportion in the community [32], while the other plentiful species do not present a significant function contribution [11], that is, the size of functional redundancy is not dependent on the amount of redundant species. This result suggests that the redundancy indices obtained using these two methods represent two different aspects of community redundancy respectively, i.e., species redundancy and functional redundancy.

### Redundancy hypothesis is applicable to diversity-stability relationships of Inner Mongolia typical steppe

The redundancy hypothesis predicts a positive impact of redundancy on stability [11]. However, the word ‘redundancy’ in its literal sense, means superfluous or unnecessary, thus the redundancy hypothesis was believed by many scholars to conflict with the biodiversity conservation hypothesis. For example, some scholars argued that the key point and priority of biodiversity protection should be focused on species with specific contributions to ecosystem functions [41, 42]; while the others believed that the diversity of biotic communities is essential for stability, thus it is not recommended to protect some species in a community while ignoring the other species [15]. Furthermore, controversial opinions still exist on whether the redundant communities are always stable and always in line with the redundancy hypothesis [6]. Some researchers believed that the redundancy and stability of the community are not always positively correlated [14, 18]. For example, Mori et al. [6] believed that functional groups with high redundancy may be more vulnerable than functional groups with low redundancy under environmental fluctuations, and thereby leading to a reduced stability in communities that consist of functional groups with high redundancy. Thus, a high redundancy is not necessary to ensure diverse responses to environmental stress and maintain the functionality under external interference [14].

This study shows that both the species redundancy and functional redundancy are positively correlated with the community stability, indicating a positive effect of redundancy on community stability, and provides some evidence to verify the redundancy hypothesis. In a functional group containing a variety of species, the species often manifest functional redundancy, and a functional group or community containing higher redundancy tends to achieve niche differentiation [11, 14] during times of external disturbance, and thereby provides an insurance effect in maintaining system stability during disturbance process.

### Functional redundancy is more important in maintaining stability compared with species redundancy

Species redundancy and functional redundancy are two features of community stability. Our results show that both the species redundancy and functional redundancy present a positive correlation with stability, with functional redundancy having a greater effect than the species redundancy. This result is reasonable, and can be explained from the calculation of the redundancy, the relationship between community complexity and stability and the effects of scale. Firstly, the species redundancy calculated here has some shortcomings because it reflects the richness of species with the same function in the community [15]. Although this method effectively differentiates the current active species and dormant species by replacing species redundancy with richness of the rest of the species beyond those with accumulative contribution rate of 80% in the community [6], avoiding the inclusion of active species into redundant species, it has shortcomings. On one hand, the calculations depend primarily on the investigator’s objective classification of the functional groups that emphasizes the morphology instead of functional properties of the species [43]. On the other hand, this method assumes all the species have equivalent positions in the community, when in fact each species may contribute to the community in significantly different ways [44]. The functional redundancy manifests a part of species diversity that has not been explained by functional diversity in the community [20], or the part of dormant functions of species in the system [6]. The functional redundancy is represented by the difference between species diversity and functional diversity. However, calculations of species diversity and functional diversity are based on parameters of individual species. Therefore, the former is more likely to describe the redundancy at functional group level, while the latter can more effectively represent the redundancy at species level [21] and thus provide more accurate redundancy information in the community. Secondly, according to McCann [45], complexity may lead to stability, but the driving force for generating this relationship is the structure and function of the system instead of the complexity itself. A small number of keystone species provide the main maintenance mechanism of functional redundancy and stability, and the role of these keystone species is the manifestation of community functions. Thirdly, there is a lack of a time-scale test. Although some species manifest intermittent redundancy in the community, species with dormant functions may respond to future disturbances [14, 46]. This time asynchronism among species, or dynamic compensation effect, is likely to provide an insurance effect on long-term stability of ecosystem [6, 17]. This study highlights the role of two kinds of redundancies on community stability at a time point, but it is not able to demonstrate the effect of species redundancy for long-term stability of the community. A long-time scale test is required to verify whether species redundancy is likely to improve the community stability.

## Supporting Information

S1 Table The minimum dataset underlying our findings. (XLSX)
